# Current Care and Investigational Therapies in Achondroplasia

**DOI:** 10.1007/s11914-017-0347-2

**Published:** 2017-02-21

**Authors:** Sheila Unger, Luisa Bonafé, Elvire Gouze

**Affiliations:** 1 Service of Genetic Medicine, Lausanne University Hospital (CHUV), Av. Pierre-Decker 2, 1011 Lausanne, Switzerland; 2 Center for Molecular Diseases, Service of Genetic Medicine, Lausanne University Hospital (CHUV), Av. Pierre-Decker 2, 1011 Lausanne, Switzerland; 3 Institute de Biologie Valrose, University. Nice Sophia Antipolis, Batiment Sciences Naturelles; UFR Sciences, Parc Valrose, 28 avenue Valrose, 06108 Nice, Cedex 2 France

**Keywords:** Achondroplasia, *FGFR3*, Treatment, Biotherapies, Clinical management

## Abstract

**Purpose of Review:**

The goal of this review is to evaluate the management options for achondroplasia, the most common non-lethal skeletal dysplasia. This disease is characterized by short stature and a variety of complications, some of which can be quite severe.

**Recent Findings:**

Despite several attempts to standardize care, there is still no widely accepted consensus. This is in part due to absence of concrete data on the incidence of sudden unexplained death in infants with achondroplasia and the best investigation for ascertaining which individuals could benefit from foramen magnum decompression surgery.

**Summary:**

In this review, we identify the different options of care and management for the various orthopedic, neurologic, and respiratory complications. In parallel, several innovative or drug repositioning therapies are being investigated that would restore bone growth but may also prevent complications. Achondroplasia is the most common non-lethal skeletal dysplasia. It is characterized by short stature and a variety of complications, some of which can be quite severe. Despite several attempts to standardize care, there is still no widely accepted consensus. This is in part due to absence of concrete data on the incidence of sudden unexplained death in infants with achondroplasia and the best investigation for ascertaining which individuals could benefit from foramen magnum decompression surgery. In this review, we identify the different options of care and management for the various orthopedic, neurologic, and respiratory complications. In parallel, several innovative or drug repositioning therapies are being investigated that would restore bone growth but may also prevent complications.

## Introduction

Achondroplasia is a rare genetic disorder for which no cure is available. This skeletal dysplasia is the most common form of short limb dwarfism and was first reported in 1878 [1]. It is estimated that it affects approximately 250,000 people worldwide [2•, 3, 4]. The incidence is estimated to be 1 in 10,000 to 30,000 live births per year [5], affecting both males and females with equal frequency.

Achondroplasia is characterized by prenatal onset of disproportionate short stature [6, 7]. Most affected children and adults enjoy good general health, but numerous neurological, orthopedic, and otolaryngologic complications can occur in this disorder, and an association with sudden infant death has been reported [8]. The phenotype of achondroplasia is caused by abnormal endochondral bone development, thus mainly affecting the growth of the long bones; the vertebrae; and several bones in the skull, including the temporal, occipital, sphenoid, and ethmoid bones. The trunk is narrow but of normal size. Adult height is 131 ± 5.6 cm in males and 124 ± 5.9 cm in females [7, 9].

Achondroplasia is caused by a single point gain-of-function mutation in the gene coding for fibroblast growth factor receptor 3 (FGFR3) [10]. It follows an autosomal dominant inheritance; though in 80% of the cases, it is a de novo mutation [11] associated with increased paternal age, relative to the general population [12–15]. In 90% of the cases, the mutation is a Gly380Arg substitution (the most common mutation is 1138G > A transition; a G > C transversion has been reported in 20% of the cases) [11, 16, 17]. Diagnosis can be suspected in utero by ultrasound and/or clinical and radiological features at birth but should be confirmed by molecular testing [18]. To help with diagnosis of skeletal dysplasia including achondroplasia, non-invasive prenatal testing is being developed [19]. The penetrance is 100% and the phenotype is already apparent at birth. Infants with achondroplasia present characteristic features with macrocephaly with frontal bossing, midface hypoplasia, and flat nasal bridge. They have short limbs with predominantly proximal (rhizomelic) shortening of the upper limbs [20], joint laxity, and a trident hand [21]. Intellect is not affected and their lifespan is close to that of the general population. Children with achondroplasia present motor delays notably to acquire standing position and walking due to muscle hypotonia and ligamentous laxity. More than 50% of the patients develop an early obesity that augments the morbidity associated with lumbar lordosis as well as the severity of sleep apnea or orthopedic complications such as *genua vara*.

Achondroplasia is a short stature condition compatible with good general health and normal life expectancy. Besides the burden of short stature, health issues may arise from complications, due to the particular anatomical features of achondroplasia.

### Current Care and Management

#### Management

Recommendations for management of children with achondroplasia have been proposed and updated over time [2•, 22, 23]. However, these guidelines are mainly based on personal experience of the authors and not on clinical studies.

Treatments aimed at correcting growth have been tried; growth hormone therapy has no significant effect on adult stature [24]. Surgical limb lengthening can improve body proportions and final height up to 20 cm but requires multiple surgeries with high rate of complications and physical burden [25]. The choice of undergoing limb lengthening depends largely on cultural and psychosocial factors.

**Fig. 1:**
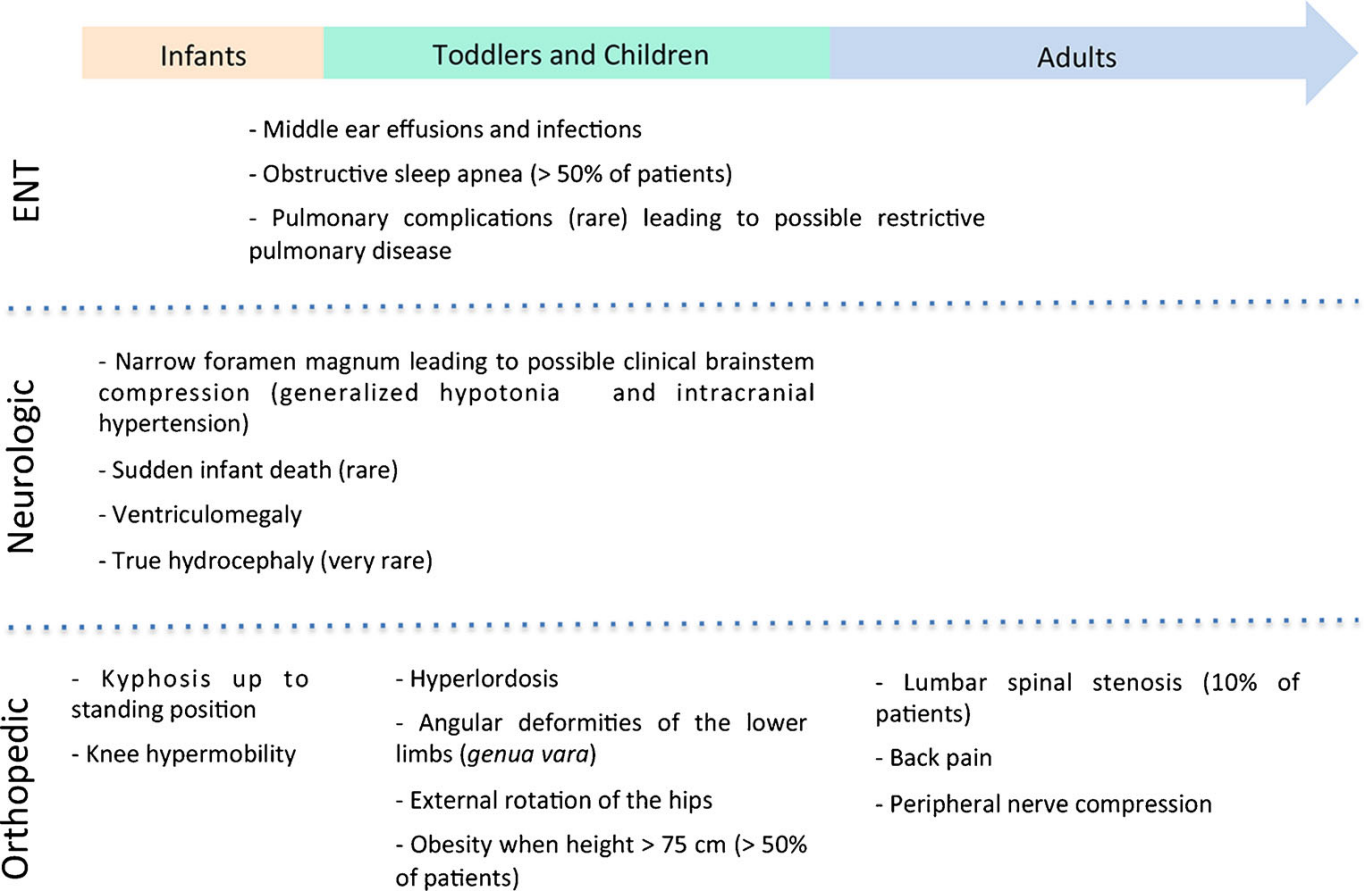
Timelines of the different complications occurring in achondroplasia patients

Current management of achondroplasia consists essentially in prevention and treatment of complications. While there is agreement on complications and their pathophysiology, there is no consensus regarding the type and frequency of surveillance because of the lack of prospective controlled clinical studies. The exact prevalence of each complication is unknown, in some cases overestimated; it is likely that the overall prevalence of complications reaches at least 10%. Figure 1 summarizes the different complications occurring in infants, toddlers and children, or adults. The most difficult task for clinicians faced with parents of a newborn with achondroplasia is to provide the right information and awareness about the condition, without overmedicalizing the infant.

#### Monitoring

Growth and development should be monitored according to norms for achondroplasia; special charts exist for height, weight, and occipital frontal circumference (OFC) [6, 22]. OFC is physiologically larger in achondroplasia and should be monitored regularly (monthly in the first year of life) in order to detect any unusual acceleration as a possible sign of hydrocephaly, a rare but potentially severe complication. Gross motor skills are often delayed [26, 27], probably due to the anatomical-biomechanical features of this condition with large head, short limbs, and joint hypermobility [28]. Fine motor and feeding skills are usually not delayed and language delay is variable. Overall, most children reach all normal developmental milestones and have no neurological or intellectual impairment; access to physiotherapy, occupational therapy, and language therapists with experience in achondroplasia may significantly improve timing of autonomy [26].

#### Complications

The most frequent complications observed in achondroplasia children are ENT issues: upper airways are anatomically smaller in achondroplasia and as a consequence, middle ear effusions and infections occur frequently, sometimes leading to conductive hearing loss and subsequent speech delay; tonsillectomy and adenoidectomy may be necessary to reduce airway obstruction, especially in the case of obstructive sleep apnea [29]. Obstructive sleep apnea is reported in >50% of patients [30], possibly because of a combination of small airways, hypotonia, and midface hypoplasia. Clinical surveillance by an ENT specialist with experience in achondroplasia is recommended particularly between 1 and 4 years of age. Pulmonary complications are rare, but restrictive pulmonary disease may occur, particularly in children living at high altitude [23].

**Fig. 2:**
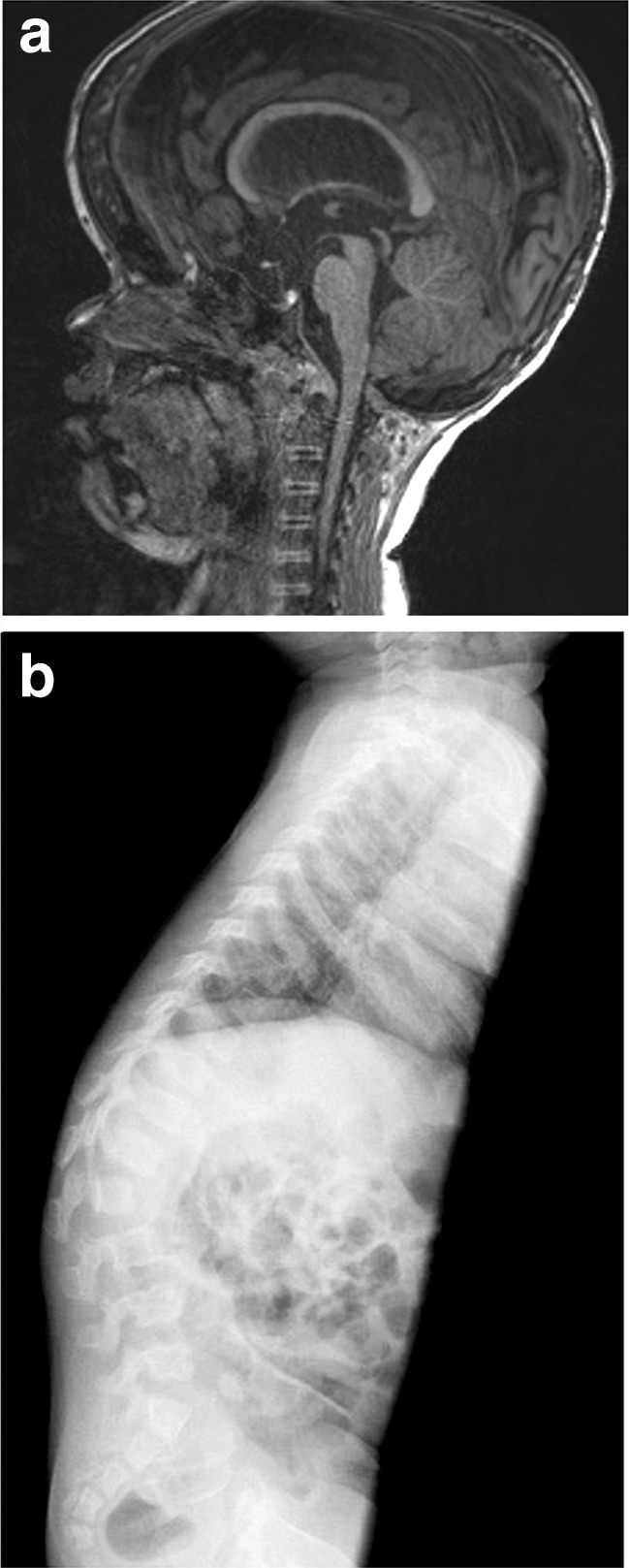
**a** MRI image of a four and a half year old boy showing a narrow foramen magnum. This finding is seen in almost all children with achondroplasia and is not by itself an indication to perform neurosurgery. **b** X-ray showing the typical configuration of the lumbar spine in achondroplasia with shortening in the AP diameter and scalloping if the posterior endplates. More significant shortening of the first lumbar vertebra is also a risk factor for developing a fixed kyphosis and would be an indication for treatment by bracing

For infants with achondroplasia, their parents, and their doctors, the most frightful complication is sudden infant death. It has been reported to occur in up to 7.5% of achondroplasia infants <1 year [31], but its incidence is probably overestimated; a more recent study reports a sixfold increase in infant mortality in achondroplasia compared to the general population [32]. It is thought to be due to spinal cord compression at the cervicomedullary junction secondary to a stenotic foramen magnum. Sleep-disordered breathing may contribute to increased mortality; however, no clear correlation has been found between central sleep apnea and foramen magnum stenosis. Always in relationship with foramen magnum stenosis, ventriculomegaly is frequent in achondroplasia infants, due to disturbed CSF circulation at the foramen magnum, but true hydrocephaly as a cause of acute intracranial hypertension seems to be much rarer [33, 34]. Some centers perform serial MRI, electrophysiological and sleep studies starting from birth and during the first year of life. However, this surveillance is not without drawbacks: brain and spinal MRI require general anesthesia and somato-sensory evoked potentials may not show changes in presymptomatic phase. In addition, all babies with achondroplasia have a narrow foramen magnum (Fig. 2a), which usually improves with age [35]. No specific marker has been identified so far to distinguish which asymptomatic patients need prophylactic decompression of the foramen magnum, and there is no evidence-based strategy; it is currently recommended that only symptomatic infants (with some clinical neurological signs and/or pathologic polysomnography) undergo brain imaging [36••]. Careful clinical surveillance by a multidisciplinary team experienced in achondroplasia may prove to be as sensible as investigations in detecting critical brainstem compression: increased generalized hypotonia, changes in osteotendinous reflexes (hyperreflexia, clonus, asymmetry), and subtle signs of intracranial hypertension (prominent fontanella, rapid increase of OFC) should be monitored, preferably by the same professional, every 2 months [36 ^••^]; family doctors should be informed about signs and symptoms to be surveyed in between the visits at the specialized center. Some centers propose sleep studies and even matrasses with detectors of sleep apnea; the utility of these measures has not been proven and the occurrence of sleep apnea appears to be only poorly correlated with foramen magnum stenosis [37, 38].

Orthopedic complications are possible at all ages. In the first year of life, some degree of kyphosis is always present at the low thoracic and lumbar region (Fig. 2b); it persists until acquirement of standing position, when it reverses to a hyperlordosis. It is recommended to avoid carrying the baby in the first months of life in baby wraps, which do not support the back and favor a kyphotic posture. Later on, the sitting position should not be imposed before the baby has enough muscle strength to sit autonomously. Bracing is not needed in all cases but may be useful to contain kyphosis when the baby is in a sitting position (meals, landau walks). Some children are particularly hypermobile and very delayed in sitting autonomously and standing. In these cases, to avoid a fixed kyphosis, appropriate bracing and/or adapted seats supporting the dorsolumbar spine should be provided. Hypermobility of the knee may also lead to instability and delayed walking but does not usually cause deformity or pain in the first years of life. Physiotherapy may be helpful in gaining correct postures and motoric strategies before free walking is achieved.

In toddlers and children, angular deformities of the lower limbs may develop with walking. Referral to orthopedic surgeons is reserved for patients with a specific problem, such as marked *genua vara*. External rotation of the hips is frequent and benign and usually resolves when the child starts walking. Obesity is an additional risk factor for orthopedic deformities and joint complications. Starting from when the child has achieved a height of 75 cm, weight/height ratio should be monitored [6] and, if necessary, nutritional counseling be offered.

In adults, the main orthopedic complication is lumbar spinal stenosis between L1 and L4, due to a narrow spinal canal and shorter vertebral arches. It can cause back pain and peripheral nerve compression. Spinal stenosis affects approximately 10% of adults with achondroplasia, and decompression surgery may be necessary in young adults [39].

#### Daily Living

Living with achondroplasia is associated with all the practical problems of short-limbed short stature in respect to architectural barriers (access to toilets, public desks, automats, public transports, etc). In addition, short arms and limited elbow extension significantly limit autonomy in personal care (dressing, personal hygiene, etc.). Occupational therapy, particularly from school age on, is helpful in finding strategies to overcome these limitations.

Social functioning is normal for most children with achondroplasia. The psychosocial burden of achondroplasia depends on several personal, familial, ethnic, and cultural factors. Some families will have a strong perception of handicap for a child with achondroplasia, while others will not perceive the condition as a problem. Educational guidance for parents of children with achondroplasia should be offered at the achondroplasia multidisciplinary clinic, to prevent overprotection, limited autonomy, and excessive anxiety. Psychological support should be offered to help acceptance of the condition, first to parents of affected infants in order to promote establishment of appropriate affective links and later to children and teenagers facing frustration and rebellion toward diversity. Support groups may also be helpful for patients and families to share experiences and reinforce positive individual resources.

### Investigational Therapies

**Fig. 3:**
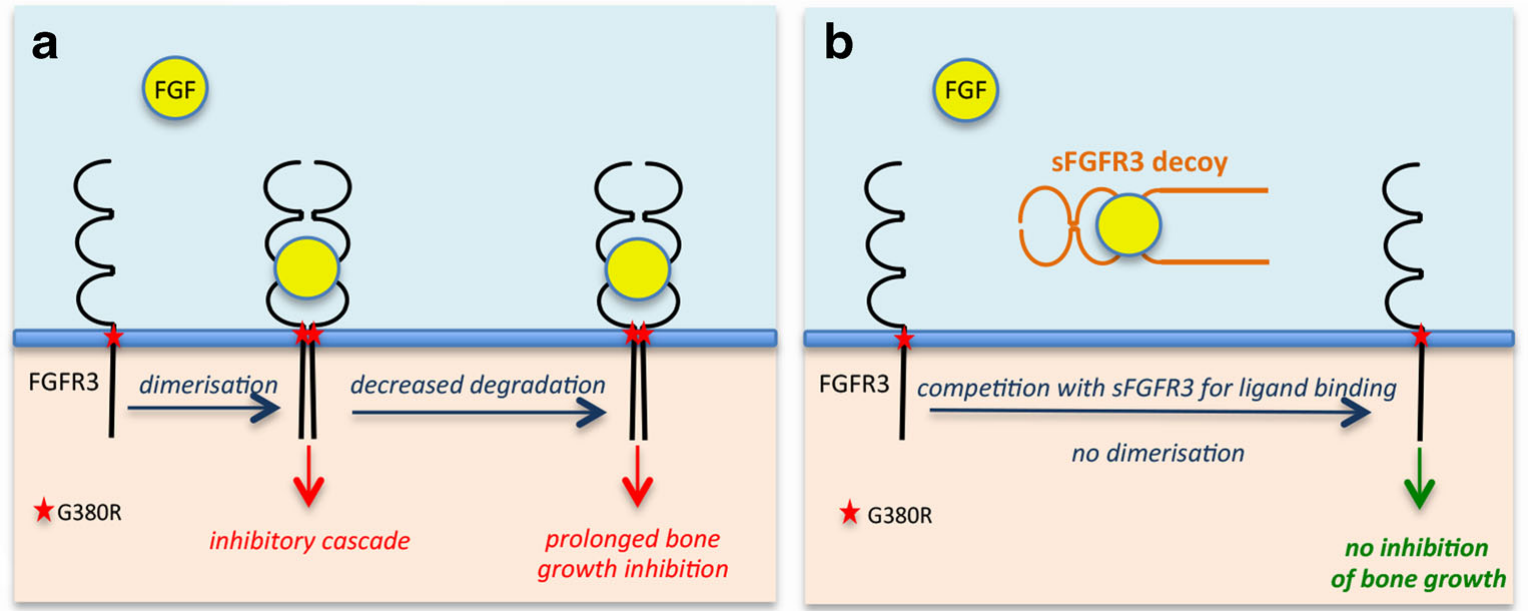
Schematic representation of FGFR3-mediated inhibition of bone growth in achondroplasia (**a**) and of the sFGFR3 decoy strategy (**b**)

Current care for achondroplasia manages the symptoms and alleviates the consequences of complications. To be effective, a new treatment approach must consider achondroplasia as a complex disease, correcting the short stature and preventing complications. Understanding the pathophysiology of the disease is essential to achieve this and correct the growth of all bones affected by the disease. As stated above, achondroplasia is caused by a mutation in the *FGFR3* gene resulting in abnormal endochondral ossification. FGFR3 signaling negatively regulates endochondral bone growth [40] by inhibiting the rate of chondrocyte proliferation and the initiation of chondrocyte hypertrophy [41] through activation of the Stat1 and MAPK pathways [42, 43]. In achondroplasia, the G380R mutation, located in the transmembrane domain of FGFR3, extends the signaling cascade resulting in prolonged bone growth inhibition (Fig. 3a). The dimerization and activation of mutant FGFR3 is ligand-dependent [44–46] and stabilizes the ligand/receptor complex by decreasing its internalization [46–48].

Several approaches have been developed to target FGFR3 within the cartilage either by blocking its activation, inhibiting downstream signaling, or increasing its turnover [49 ^•^]. Among them, we have recently evaluated the use of a FGFR3 decoy receptor (sFGFR3) to avoid the FGF ligand from binding to the receptor, thus preventing activation of the intracellular signaling directly downstream of mutant FGFR3 resulting in bone growth activation (Fig. 3b) [45]. The sFGFR3 decoy lacks the transmembrane domain and is thus secreted from the cells, unable to activate the signaling cascade [50]. sFGFR3 was administered subcutaneously for 3 weeks to mice carrying the G380R mutation (*Fgfr3* ^*ach/+*^ mice) and was able to penetrate the growth plate cartilage. At a dose of 2.5 mg/kg twice weekly from age 3 days to 3 weeks, sFGFR3 treatment was effective at restoring normal body, tail, and long bone lengths in treated *Fgfr3* ^*ach/+*^ mice. Interestingly, following treatment, significant reduction in respiratory failure and spinal compression were observed. Treated *Fgfr3* ^*ach/+*^ mice also showed normal rib cage development and decreased vertebrae and skull deformities. No obvious toxicity was observed and treatment did not affect reproduction. One unexpected advantageous side effect was the increase in pelvis size in primiparous-treated *Fgfr3* ^*ach/+*^ females resulting in normal size litters. If this translates into humans, this would be a significant advantage, as during delivery achondroplasia patients must undergo C-section due to small pelvis size. The decoy approach shows promise as a potential treatment for achondroplasia restoring not only the stature but also preventing most of the complications due to the characteristic features of achondroplasia.

A 12 amino acid peptide (called P3) binding with high affinity to the extracellular domain of FGFR3 has been evaluated in mice with thanatophoric dysplasia type II (TDII), a more severe form of FGFR3-related skeletal dysplasia [51]. In vitro, P3 treatment restored chondrocyte proliferation and differentiation by decreasing the MAPK signaling cascade. When administered to neonatal TDII pups, animals survived and treatment partially rescued endochondrial bone growth. Interestingly, this treatment had positive effects on long bones but also rescued neonatal lethality potentially preventing some of the complications caused by spinal and skull deformities.

In 2014, Yamashita et al. evaluated the use of statin to rescue bone growth in achondroplasia and thanatophoric dysplasia type I (TDI) [52••]. The rationale for repositioning this pleiotropic drug is that it appears to stimulate bone growth through anabolic effects on chondrocytes [53–55]. While the exact mechanism of action is unknown, it is suspected that statin treatment may accelerate FGFR3 degradation on chondrocytes [52••, 56]. In this study, the authors first develop a cell-based system using induced pluripotent stem cells (iPSCs) generated from dermal fibroblasts of achondroplasia and TDI patients. Following reprogramming of these cells and differentiation into chondrocytes, this cell culture assay displays similarities with patient chondrocytes with decreased chondrogenic potential that was rescued by lovastatin treatment. In vivo, daily intraperitoneal injections of 1 mg/kg rosuvastatin from 3 to 6 weeks of age partially restored limb and skull lengths of *Fgfr3* ^*ach/+*^ mice showing potential therapeutic effects of statins. However, while there are abundant data on the use of statin in humans, little is known about safety in children.

Another repositioning approach has been evaluated recently using meclozine, an over-the-counter antihistamine drug used for motion sickness. The authors initially showed in vitro effects of meclozine on chondrocyte proliferation and differentiation [57]. Transgenic *Fgfr3* ^*ach/+*^ mice orally treated with meclozine showed an improvement in the dwarf phenotype with an increase in bone length [58]. The specific mechanism remains unknown, and the safety profile of chronic administration of meclozine in children has yet to be evaluated.

Small molecule approaches targeting tyrosine kinase domains have been explored for over a decade in anticancer therapies [59]. Their application to skeletal dysplasia appears promising at rescuing the achondroplasia phenotype in vitro and in explant culture systems [60–62]. However, the relevance of their in vivo application is unclear because of their absence of selectivity for FGFR3 [60], potentially inhibiting the activities of other FGFR and other tyrosine kinases, inducing multiorgan toxicity [62]. Additional development is needed to further improve the specificity.

Biomarin Pharmaceuticals is currently conducting a phase 2 clinical trial on children with achondroplasia using vosoritide, a C-type natriuretic peptide (CNP) analog [49•]. The CNP approach has been evaluated for nearly two decades now and acts indirectly on FGFR3 signaling though the MAPK signaling pathway not the Stat1 cascade [63, 64]. Skeletal phenotype was rescued in double transgenic mice overexpressing both the mutant FGFR3 receptor and CNP in growth plate chondrocytes [65]. Following chronic infusion of CNP, mice with achondroplasia had longer long bones but also larger foramen magnum, suggesting a potential treatment effect on the occurrence of the complications [66, 67]. Biomarin is now using an analog with a prolonged half-life that proved to be effective at increasing bone length in mice and growth plate width in monkey [68]. The initial phase 2 results are very encouraging demonstrating a favorable safety profile and an increase in 50% of the annualized growth velocity following daily subcutaneous administration of 15 g/kg vosoritide for 12 months compared to the pretreatment growth velocity [69]. The body proportions, calculated as the ratio of upper body length to leg length, which is higher in children with achondroplasia, were not improved by vosoritide treatment. In a prospective observational study, Olney et al. recently showed that CNP plasma levels are elevated in achondroplasia patients suggesting that these patients may have a natural resistance to CNP [70]. Further investigation would be necessary to clarify this as it could interfere with long-term treatment efficacy of vosoritide.

## Conclusion

In spite of being the most common form of skeletal dysplasia, there exists no clear consensus on care and management for children and adults with achondroplasia. Developing a routine healthcare is of high importance and would greatly benefit some patients who tend to be overmedicalized or are far removed from specialized centers. A natural history multicenter study has been very recently started to increase the “collaboration among researchers to gather similarly affected patients to answer common clinical research questions”. This type of initiative is essential to improve care and management of these patients. In parallel, the development of biotherapies directly targeting FGFR3 could resolve these issues. However, in addition to rescuing skeletal growth, these therapies should address all of the other (or at least some) clinical aspects of achondroplasia thus preventing the development of complications.
